# Analysis of Eye Movements in Adults with Spinal Muscular Atrophy

**DOI:** 10.3390/medicina61040571

**Published:** 2025-03-23

**Authors:** Marek Krivošík, Zuzana Košutzká, Marián Šaling, Veronika Boleková, Rebeka Brauneckerová, Martin Gábor, Peter Valkovič

**Affiliations:** 1 2nd Department of Neurology, Faculty of Medicine, Comenius University Bratislava, 813 72 Bratislava, Slovakia; zuzanakosutzka@gmail.com (Z.K.); marian.salingg@gmail.com (M.Š.); veronika.bolekova@gmail.com (V.B.); gabor.physio@gmail.com (M.G.); 2 Faculty of Psychology, Institute of Clinical Psychology, Pan-European University, 821 02 Bratislava, Slovakia; 3 Department of Physiotherapy, Balneology and Medical Rehabilitation, Slovak Medical University, 833 03 Bratislava, Slovakia; brauneckerova.r@gmail.com; 4 Department of Physiotherapy, Balneology and Medical Rehabilitation, University Hospital Bratislava, 821 01 Bratislava, Slovakia; 5 Institute of Normal and Pathological Physiology, Centre of Experimental Medicine, Slovak Academy of Sciences, 813 71 Bratislava, Slovakia

**Keywords:** spinal muscular atrophy, smooth pursuit, saccades, eye movements

## Abstract

*Background and Objectives*: Spinal muscular atrophy (SMA) is a progressive, autosomal recessive, rare neuromuscular disorder caused by a genetic defect in the *SMN1* gene, where the *SMN2* gene cannot sufficiently compensate. Patients experience progressive and predominantly proximal muscular weakness and atrophy. Oculomotor disorders are currently not regarded as a typical feature of SMA. The aim of this study was to determine whether oculomotor abnormalities are present in subjects with SMA and to assess a potential relationship between the oculomotor parameters and disease duration. *Materials and Methods*: An analysis of 15 patients with SMA type 2 and type 3 and 15 age-matched healthy controls was conducted. The oculomotor performance, including the analysis of smooth pursuit velocity gain and saccades parameters (latency, velocity, accuracy) in the horizontal and vertical directions, was compared between both groups. *Results*: The analysis of smooth pursuit gain in the participants revealed a marginally significant reduction between the SMA patients and the healthy controls in the horizontal direction at a frequency of 0.2 Hz (*p* = 0.051), but no significant differences were observed at any other frequency or direction. The vertical velocity of the saccade eye movements of the SMA patients was increased compared with the healthy subjects, which was statistically significant for the amplitude of ±10° (*p* = 0.030), but not for the amplitude of ±16.5° (*p* = 0.107). The horizontal saccade latency, saccade velocity and saccade accuracy did not differ significantly between the SMA patients and the controls. None of the oculomotor parameters were associated with disease duration. *Conclusions*: While certain oculomotor abnormalities, such as increased vertical saccade velocity, were observed in the SMA patients, these findings do not indicate a defining role of oculomotor impairment in SMA pathology or its clinical characteristics.

## 1. Introduction

Spinal muscular atrophy (SMA) is a rare, debilitating neuromuscular disease, resulting in progressive muscle weakness and atrophy caused by a homozygous deletion or mutation of the Survival Motor Neuron 1 (*SMN1*) gene, which results in the loss of SMN protein expression and the subsequent degeneration of spinal cord and brainstem motor neurons. The absence of the *SMN1* gene on both alleles (chromosome 5q13.2) is responsible for approximately 95% of cases of SMA. In the remaining 5%, individuals carry another pathogenic variant in trans with a deletion of the second SMN1 allele or, in rare instances, with a biallelic occurrence of other pathogenic variants in SMN1 [1]. The severity of SMA generally correlates with number of copies of the *SMN2* gene, which produces the SMN protein with reduced function, partially compensating for the *SMN1* gene mutation or deletion. SMA is classified into four types based on the age at onset and maximum motor ability, ranging from the most severe, SMA type 1, to the mildest, SMA type 4. Patients with SMA type 2 (accounting for 20–29% of SMA cases) have an intermediate form of SMA, with symptoms including muscle weakness and respiratory insufficiency that first appear between 6 and 18 months of age. Approximately 13–15% of patients with SMA have SMA type 3, with an age of onset ranging from 18 months to 20 years. Patients with SMA type 3 may achieve the ability to stand or walk without support (walkers), though this ability may be lost with disease progression [2]. Considering the current knowledge, SMA is regarded not merely as a motor neuron disease (MND), but as a multisystem disease affecting many different tissues and organs [3].

The primary function of eye movement is to maintain the image of an object in the fovea, thereby preventing the image from slipping on the retina. The accurate evaluation of eye movements provides invaluable insight into the functions of the oculomotor system and facilitates the identification of any abnormalities [4]. Prior to the advent of genetic testing, isolated clinical reports indicated that abnormal eye movements may occur in patients with SMA [5,6,7]. A paper by Anagnostou et al. (2021) demonstrated that adult patients with SMA type 2 and type 3 perform fast and accurate horizontal saccades without evidence of extraocular muscle weakness [8]. These quantitative oculomotor data corroborate the clinical experience that neuro-ophthalmic signs are uncommon in SMA. If present, they should prompt the consideration of an alternative neuromuscular disorder. Consequently, the oculomotor phenotype is currently not regarded as a typical feature of SMA types 2 and 3, and is not considered a biomarker for disease monitoring. The ocular motor neurons, which innervate the extraocular muscles and thus control the movement of the eyes, appear consistently resistant in SMA. This is also evidenced using ocular tracking devices as a communication tool for patients with SMA type 1 [9]. The objective of this research project is to determine the diagnostic or physiological relevance of oculomotor abnormalities in subjects with SMA and to assess the potential relationships with clinical characteristics. The metrics of both saccadic and smooth pursuit eye movements will be analyzed using video-oculography in both the horizontal and vertical directions.

## 2. Materials and Methods

The study population comprised 15 consecutively recruited patients with genetically confirmed SMA. Patients were excluded if they had another neurodegenerative disease, a history of ocular disorders or diseases of the vestibular system that may affect eye movements, or were taking medications that could affect eye movements, including benzodiazepines, other stimulants or suppressive agents. None of the patients exhibited severe respiratory deficits or were treated with non-invasive ventilation. Furthermore, 15 healthy age-matched control subjects with no history or clinical manifestations of any neurological disorders and no familial relationships with the gene carriers were included. The study was conducted in accordance with the Declaration of Helsinki and the local ethics committee (Academic Derer’s University Hospital, Bratislava, Slovakia) approved study protocol No. 14/2024. The measurement and data collection process was conducted from 1 April to 30 August 2024.

Demographic and clinical data were collected at the baseline assessment. To ascertain the length of time that the disease had been present, the interval between the initial onset of symptoms and the date of oculomotor assessment was calculated. The patients with SMA were divided into two subgroups according to the age of onset of the symptoms. The first group comprised patients with a disease onset before 18 months of age (SMA type 2), and the second group comprised patients with a disease onset from 18 months of age to 20 years (SMA type 3). Patients with SMA type 4 (adult-onset) were not included in the study. The number of SMN2 copies, a known modifier of disease severity, was determined in each patient [10]. To evaluate the severity of the disease, two certified physiotherapists employed the Revised Upper Limb Module (RULM) scale in the patients with SMA type 2 and the Hammersmith Functional Motor Scale Expanded (HFMSE) scale in the patients with SMA type 3. The RULM is a tool used to assess upper limb motor function and performance in activities of daily living. The scale comprises 20 items, with a maximum score of 37 points, whereby higher scores indicate superior upper limb function [11]. The HFMSE is a 33-item disease-specific scale that assesses gross motor skills. An individual can achieve a maximum score of 66, with higher scores indicating superior motor function [12].

Eye movements were recorded using a video-based eye movement tracking system (VNG-Synapsys^®^, Marseille, France). The stimuli were viewed binocularly in a room with low illumination, and the right eye’s movements were recorded. The dominance of the eye was not established. The algorithms used to calculate the eye position were based on determining the pupil center. Prior to data collection, the device was calibrated for each participant by recording fixations in the central and eccentric positions. The participant’s head was stabilized on a support, with the temporal bones supported on both sides. The calibration demonstrated that all participants could see the targets with sufficient clarity to locate them without difficulty. The visual targets were displayed on a screen with a width of 228 cm and a height of 169 cm, situated at an eye distance of 285 cm. The maximum horizontal angle was 44°, while the maximum vertical angle was 32°.

The following oculomotor paradigms were conducted in a consistent chronological order for all participants, commencing with the horizontal eye movement recordings and subsequently progressing to the vertical eye movement recordings. These paradigms encompassed smooth pursuit and saccadic movements. Prior to commencing each test, the investigator provided a detailed explanation of the task, and the subjects were asked to confirm that they fully understood the requirements of the test. If the subject did not comprehend the instructions, they were repeated, and exemplar trials were presented.

Smooth pursuit: Following an initial fixation period of two seconds, the smooth pursuit was moved sinusoidally in three cycles at frequencies of 0.2 Hz, 0.3 Hz and 0.45 Hz in both the horizontal and vertical directions. The amplitude in the horizontal direction (right and left) was set at ±20° from the central position, with a subsequent ±15° deviation in the vertical direction (up and down). The participants were required to perform smooth pursuit movements for a period of 30 s along both axes. The gain (velocity increment), defined as the relationship between the eye velocity and target velocity at a given time, was tracked during the smooth pursuit movements, and recorded for the left and right directions in the horizontal plane and for the up and down directions in the vertical plane. It decreased rapidly at higher velocities and declined gradually with advancing age [4].

Saccades: All subjects demonstrated the ability to follow visually guided horizontal and vertical saccades in response to the stimuli. Horizontal saccades with an amplitude of 20° within ±10° and 40° within ±20° and vertical saccades evoked by the stimuli with an amplitude of 20° within ±10° and 33° within ±16.5° from the central position were observed. The subjects were required to complete a 40 s test in response to a stimulus of each magnitude along both axes. The presentation of the targets was conducted in a pseudorandom order. The patients were encouraged to follow the target jumps. The following saccadic parameters were analyzed: latency, accuracy and velocity [4]. Saccadic latency is defined as the duration between the onset of a stimulus and the initiation of the saccade. Saccade accuracy is the ratio between the initial saccade amplitude and the target amplitude, and this measure indicates how accurately a saccadic movement landed on the target stimuli. The norms for saccade accuracy are conventionally set at about 90–125%. Saccade velocity is calculated by taking the first derivative of the time series of gaze position data. The average saccadic velocity is the mean of the velocities over the duration of a saccade, and the norms for the velocities depend on the amplitude.

After testing for normal distribution, Mann–Whitney U tests were employed for parameter comparisons between the patients and controls. Differences within the patient subgroups were also assessed using the Mann–Whitney U test. Due to the limited sample size of the SMA patients, subgroup analyses based on the SMA type were not performed to ensure statistical validity. Spearman’s correlation was used to test the association of the smooth pursuit and saccadic parameters with disease duration. Significance was set at 0.05.

## 3. Results

Fifteen adult patients with genetically confirmed SMA (10 females, 5 males, age ±SD 34.1 ± 9 years, mean disease duration 29.9 ± 10.3 years) were included in the study. Two patients (13.3%) had a first-degree relative with SMA. All 15 patients exhibited a deletion of exon 7 in the *SMN1* gene, with three copies of the *SMN2* gene present in six patients (40%) and four copies of the *SMN2* gene present in nine patients (60%). The control group comprised 15 individuals with no history of neurological or ophthalmic disease (9 females, 6 males; mean age ±SD 33.5 ± 8.4 years). The disease severity in the SMA subjects as assessed by the RULM for SMA type 2 ranged from 0 to 22 (median, 11), and by the Hammersmith Functional Motor Scale Expanded for SMA type 3 ranged from 32 to 54 (median, 42.5). A summary of the demographic and clinical characteristics of the SMA patients and control subjects is presented in Table 1.

There were no significant differences between the rightward and leftward or between the upward and downward smooth pursuit gains in either the patients or the controls. Therefore, the data were pooled for further analysis.

The horizontal smooth pursuit gain in response to a 0.2 Hz stimulus frequency was 0.88 ± 0.10 in the SMA patients and 0.94 ± 0.08 in the control subjects (*p* = 0.051). A *p*-value that is slightly above 0.05 is often reported as “marginally significant”, which suggests that a subtle effect may still be present, but no significant differences in horizontal smooth pursuit gain were observed between the groups at any other frequency. The vertical smooth pursuit gain in response to a 0.2 Hz stimulus frequency was 0.75 ± 0.13 in the SMA patients and 0.76 ± 0.12 in the control subjects (*p* = 0.836). A summary of the details can be found in Table 2.

A total of 20% (3/15) of the SMA patients and 6.7% (1/15) of the control subjects showed abnormal cogwheeling during smooth pursuit; the difference between the groups was not significant.

The horizontal saccade latency, velocity and accuracy revealed no significant differences between the SMA patients and the healthy controls (Table 3). Horizontal saccades in response to a stimulus of 10° yielded a velocity of 360.93°/s ± 89.29 in the SMA patients and 319.96 ± 61.37 in the control subjects (*p* = 0.076).

Regarding the vertical direction, the saccades in response to a stimulus of 10° yielded a velocity of 327.10 ± 66.34°/s in the SMA patients and 280.88 ± 32.07°/s in the control subjects (*p* = 0.030), whilst the saccades in response to a stimulus of 16.5° measured 364.10 ± 102.59°/s in the SMA subjects and 419.58 ± 74.83°/s in the control subjects (*p* = 0.107) (Table 4).

The Spearman’s correlation coefficient between the saccadic and smooth pursuit eye movements and disease duration in the cohort was evaluated, and the results were not statistically significant (Supplementary Tables S1–S3).

## 4. Discussion

Although oculomotor impairment is not a defining characteristic of SMA, ocular motility in SMA has not been subjected to systematic analysis due to the rarity of the condition. Accordingly, the present study investigated a range of ocular motor functions, including vertical and horizontal saccades and smooth pursuit of vertical and horizontal gaze.

Our study confirmed that patients with SMA (type 2 and 3) have faster vertical velocities of the saccades than seen in healthy controls. It is beyond doubt that saccadic velocity represents the most sensitive indicator of extraocular muscle weakness [8]. While some studies have reported comparable saccadic parameters in individuals with MND and control groups, others have observed differences in duration, latency, velocity and the overall incidence of abnormalities [13]. Importantly, different MND phenotypes were selected in different studies. Only one published study observed an increase in peak velocity in individuals with amyotrophic lateral sclerosis (ALS) compared to a control group [14]. Excitatory burst neurons, which produce the pulse of innervation necessary for vertical saccades, are located in the rostral interstitial nucleus of the medial longitudinal fasciculus, while the neural integrators, responsible for maintaining the eyes at the new position, are found in the interstitial nucleus of Cajal [15]. To date, neuropathology studies of SMA have focused mostly on spinal ventral horn motor neurons, the proprioceptive synaptic inputs to motor neurons and the synapses of motor neurons onto muscles—known as neuromuscular junctions. Potentially disease-related dysfunction in the brain neural circuits for motor and ocular control (cerebral cortex, cerebellum, midbrain and lower brainstem) has been seldom studied [16]. Consequently, there is an absence of data regarding the function of the brainstem in regulating eye movements in patients diagnosed with SMA.

A comparison of saccadic parameters in the horizontal direction in patients with SMA and healthy controls revealed no significant differences in our cohort. Our findings are in accordance with a previously reported study with a similar patient group [8].

The analysis of smooth pursuit gain in the participants revealed a marginal significant reduction between the SMA patients and the healthy controls in the horizontal direction at a frequency of 0.2 Hz, but no significant differences were observed at any other frequency or direction. Smooth pursuit movement plays a role in stabilizing the image of a small moving object on the fovea. The initiation and maintenance of this movement depends on pathways involving the cortical and visual areas and their projections to the nuclei of the pons and cerebellum. The cortical areas project via the pons to the cerebellum, sending signals via the deep cerebellar and vestibular nuclei to oculomotor neurons in the brainstem to initiate tracking [17]. The research into smooth pursuit in people with MND revealed an increased frequency of saccadic intrusions (anticipatory saccades and square wave jerks), a reduction in gain and abnormal cogwheel eye movements (the interruption of smooth pursuit movements by catch-up saccades, resulting in jerky, uneven eye movements) [13].

The selective resistance of ocular motoneurons to the effects of neurodegeneration remains an area of ongoing research. A microarray analysis of human gene expression profiles of ocular motoneurons versus skeletal motoneurons revealed some expected differences. These included an up-regulation of genes concerned with the mitochondrial firing rate (reflecting a high discharge rate) and HOX genes (important for the rostral–caudal migration of cells during embryogenesis) [18]. It is pertinent to consider the preservation of ocular motoneurons in ALS in the context of the up-regulation of specific glutamate and γ-aminobutyric acid (GABA) receptor units. The up-regulation of ubiquitin-proteases, which degrade proteins, may contribute to the ability of ocular motoneurons to resist degeneration. Notably, the neurofilament light subunit and synaptophysin are maintained at the neuromuscular junctions of the extraocular muscles in ALS, but not in the limb muscles [19]. The Wnt proteins, which play a crucial role in neuromuscular development and regeneration, are expressed at higher levels in extraocular than in skeletal muscles in both normal subjects and ALS patients [20]. Additionally, extraocular muscle fibers in ALS exhibit only minor alterations in the fiber type and isoforms of myosin heavy chain [21]. While these findings offer significant insights into the mechanisms underlying the selective resistance of ocular motoneurons in ALS, comparable data regarding SMA remain scarce to date.

Nonetheless, research has demonstrated that oculomotor functioning can be compromised in cases of ALS. Studies have investigated alterations in oculomotor control across the spectrum of ALS stages, ranging from the early to advanced stages. Impairments in smooth pursuit eye movements and reduced velocity in prosaccades have been documented in the early to middle ALS stages and in ALS patients with bulbar onset [22]. However, oculomotor research in the advanced stages remains limited. A paper by Aust et al. (2024) demonstrated that patients with advanced ALS showed significant deterioration in oculomotor functions. This deterioration was characterized by longer latencies in visually guided prosaccades, particularly in the vertical compared to the horizontal prosaccades, more frequent hypometria compared to healthy subjects, generally less accurate prosaccades and severe impairments in smooth pursuit eye movements, including reduced gain and increased catch-up saccades [23]. These oculomotor deficits may negatively impact the effectiveness of eye-tracking communication systems, which are crucial for patients with severe motor impairment.

Eye-tracking devices are widely utilized as an alternative means of communication, particularly for patients with conditions such as SMA type 1, where verbal and gestural communication is severely limited [9,24]. The ability to maintain a stable ocular fixation is especially critical for patients relying on gaze-controlled communication devices, as any impairment in fixation is expected to significantly impact their quality of life.

Despite the lack of comparative studies on the eye movement differences between SMA and ALS, the findings from individual studies conducted at the advanced stages of both diseases indicate some disparities. Specifically, preserved eye movements are observed in SMA, irrespective of the type and duration of the disease, in contrast to the impaired eye movements in the advanced stages of ALS.

The principal limitation of this study is the complexity of the disease, which has resulted in smaller sample sizes, thereby reducing the study’s overall power. The subsequent stage of the study will be to assess the ocular motor systems of a larger cohort of patients with SMA.

## 5. Conclusions

The current findings, derived from an assessment of the smooth pursuit and saccadic eye movements in adult patients with SMA types 2 and 3, suggest that these movements are not suitable as clinical biomarkers for disease monitoring. However, they may hold value in aiding differential diagnosis by distinguishing SMA from other neurological conditions affecting ocular motility. This underscores the importance of exploring alternative biomarkers for SMA. Further research is needed to validate these findings and expand their clinical implications.

## Figures and Tables

**Table 1:** Demographic and clinical characteristics of SMA patients and control subjects.

	SMA (n = 15)	Control Subjects (n = 15)	*p*-Value
Age, y	34.1 ± 9.0	33.5 ± 8.4	0.506
Sex, male/female	5 (33.3%)/10 (66.6%)	6 (40%)/9 (60%)	
Disease duration, y	29.9 ± 10.3		
	SMA Type 2 (n = 7)	SMA Type 3 (n = 8)	
Age, y	35.6 ± 9.6	32.8 ± 8.9	
Disease duration, y	32 ± 9.6	22.5 ± 9.9	
Disease severity			
RULM, range, median	0–22; 11	-	
HFMSE, range, median	-	32–54; 42.5	
SMN2 copy number			
3 copies	5 (71%)	1 (12.5%)	
4 copies	2 (29%)	7 (87.5%)	
SMA functional status			
Sitter	7 (100%)	2 (25%)	
Walker	0 (0%)	6 (75%)	

**Table 2:** Comparison of smooth pursuit gain between SMA patients and control subjects.

	SMA (n = 15)	Control Subjects (n = 15)	*p*-Value
Horizontal gain 0.2 Hz	0.88 ± 0.10	0.94 ± 0.08	0.051
Horizontal gain 0.3 Hz	0.85 ± 0.13	0.90 ± 0.09	0.181
Horizontal gain 0.45 Hz	0.74 ± 0.15	0.81 ± 0.13	0.369
Vertical gain 0.2 Hz	0.75 ± 0.13	0.76 ± 0.12	0.836
Vertical gain 0.3 Hz	0.69 ± 0.14	0.72 ± 0.11	0.927
Vertical gain 0.45 Hz	0.62 ± 0.16	0.56 ± 0.13	0.333

Note: Data expressed as mean ± standard deviation.

**Table 3:** Horizontal saccade parameters for SMA patients and control subjects.

	SMA (n = 15)	Control Subjects (n = 15)	*p*-Value
Latency, ms			
Target at 10°	263.73 ± 31.44	261.31 ± 22.44	0.872
Target at 20°	277.40 ± 30.94	265.69 ± 28.21	0.357
Velocity, °/s			
Target at 10°	360.93 ± 89.29	319.96 ± 61.37	0.076
Target at 20°	479.97 ± 89.82	439.12 ± 65.16	0.134
Accuracy, %			
Target at 10°	102.07 ± 15.70	97.35 ± 4.11	0.278
Target at 20°	96.00 ± 3.43	92.42 ± 5.39	0.146

Note: Data expressed as mean ± standard deviation.

**Table 4:** Vertical saccade parameters for SMA patients and control subjects.

	SMA (n = 15)	Control Subjects (n = 15)	*p*-Value
Latency, ms			
Target at 10°	287.93 ± 40.54	287.31 ± 38.47	0.836
Target at 16.5°	287.73 ± 34.15	285.92 ± 36.82	0.908
Velocity, °/s			
Target at 10°	327.10 ± 66.34	280.88 ± 32.07	**0.030**
Target at 16.5°	364.10 ± 102.59	419.58 ± 74.83	0.107
Accuracy, %			
Target at 10°	96.20 ± 11.24	94.46 ± 7.52	0.764
Target at 16.5°	90.07 ± 12.41	91.39 ± 5.04	0.518

Note: Data expressed as mean ± standard deviation; *p*-values marked with bold indicate statistically significant differences between groups.

## Data Availability

The original contributions presented in the study are included in the article; further inquiries can be directed to the corresponding authors.

## Supplementary Materials

The following supporting information can be downloaded at: https://www.mdpi.com/article/10.3390/medicina61040571/s1, Table S1 The correlation between smooth pursuit gain and disease duration. Table S2 The correlation between horizontal saccade parameters and disease duration. Table S3 The correlation between vertical saccade parameters and disease duration.
