# Supplementary material for: Analysis of Eye Movements in Adults with Spinal Muscular Atrophy
Source: Medicina (Kaunas). 2025 Mar 23;61(4):571. doi: 10.3390/medicina61040571 (PMC12028716; doi:10.3390/medicina61040571)
Supplement: Supplementary file 1 [file medicina-61-00571-s001.zip › medicina-3428279-supplementary.pdf]

Table S1 The correlation between smooth pursuit gain and disease duration

	r_s	*p*-Value
**Horizontal gain 0.2Hz**	0.018	0.950
**Horizontal gain 0.2Hz**	0.116	0.680
**Horizontal gain 0.2Hz**	0.150	0.593
**Vertical gain 0.2Hz**	0.199	0.478
**Vertical gain 0.3Hz**	0.181	0.519
**Vertical gain 0.45Hz**	0.097	0.732

r_s—Spearman’s correlation coefficient

Table S2 The correlation between horizontal saccade parameters and disease duration

	r_s	*p*-Value
**Latency, ms**		
**Target at 10°**	-0.036	0.899
**Target at 20°**	-0.132	0.638
**Velocity, °/s**		
**Target at 10°**	0.022	0.937
**Target at 20°**	-0.122	0.666
**Accuracy**		
**Target at 10°**	0.444	0.097
**Target at 20°**	0.178	0.527

r_s—Spearman’s correlation coefficient

Table S3. The correlation between vertical saccade parameters and disease duration

	r_s	*p*-Value
**Latency, ms**		
**Target at 10°**	-0.073	0.795
**Target at 16.5°**	-0.027	0.924
**Velocity, °/s**		
**Target at 10°**	-0.055	0.844
**Target at 16.5°**	0.023	0.934
**Accuracy**		
**Target at 10°**	0.343	0.210
**Target at 16.5°**	-0.017	0.952

r_s—Spearman’s correlation coefficient.
